# Multi-modal analysis of inflammation as a potential mediator of depressive symptoms in young people with HIV: The GOLD depression study

**DOI:** 10.1371/journal.pone.0298787

**Published:** 2024-02-22

**Authors:** Arish Mudra Rakshasa-Loots, Shalena Naidoo, Thandi Hamana, Busiswa Fanqa, Kaylee S. van Wyhe, Filicity Lindani, Andre J. W. van der Kouwe, Richard Glashoff, Sharon Kruger, Frances Robertson, Simon R. Cox, Ernesta M. Meintjes, Barbara Laughton

**Affiliations:** 1 Family Centre for Research with Ubuntu (FAMCRU), Tygerberg Hospital, Department of Paediatrics and Child Health, Stellenbosch University, Cape Town, South Africa; 2 Edinburgh Neuroscience, School of Biomedical Sciences, The University of Edinburgh, Edinburgh, United Kingdom; 3 Division of Biomedical Engineering, Biomedical Engineering Research Centre, Department of Human Biology, Faculty of Health Sciences, University of Cape Town, Cape Town, South Africa; 4 ACSENT Lab, Department of Psychology, University of Cape Town, Cape Town, South Africa; 5 A.A. Martinos Center for Biomedical Imaging, Massachusetts General Hospital, Boston, MA, United States of America; 6 Department of Radiology, Harvard Medical School, Boston, MA, United States of America; 7 Division of Medical Microbiology, Stellenbosch University, Cape Town, South Africa; 8 National Health Laboratory Service (NHLS), Tygerberg Business Unit, Cape Town, South Africa; 9 Neuroscience Institute, University of Cape Town, Cape Town, South Africa; 10 Cape Universities Body Imaging Centre, Cape Town, South Africa; 11 Lothian Birth Cohorts group, Department of Psychology, The University of Edinburgh, Edinburgh, United Kingdom; Brighton and Sussex Medical School, UNITED KINGDOM

## Abstract

People living with HIV are at three times greater risk for depressive symptoms. Inflammation is a notable predictor of depression, and people with HIV exhibit chronic inflammation despite antiretroviral therapy. We hypothesised that inflammatory biomarkers may mediate the association between HIV status and depressive symptoms. Participants (*N* = 60, 53% girls, median [interquartile range (IQR)] age 15.5 [15.0, 16.0] years, 70% living with HIV, of whom 90.5% were virally-suppressed) completed the nine-item Patient Health Questionnaire (PHQ-9). We measured choline and myo-inositol in basal ganglia, midfrontal gray matter, and peritrigonal white matter using magnetic resonance spectroscopy, and 16 inflammatory proteins in blood serum using ELISA and Luminex™ multiplex immunoassays. Using structural equation mediation modelling, we calculated standardised indirect effect estimates with 95% confidence intervals. Median [IQR] total PHQ-9 score was 3 [0, 7]. HIV status was significantly associated with total PHQ-9 score (*B* = 3.32, *p* = 0.022). Participants with HIV showed a higher choline-to-creatine ratio in the basal ganglia than those without HIV (*β* = 0.86, *p*_FDR_ = 0.035). In blood serum, participants with HIV showed higher monocyte chemoattractant protein-1 (MCP-1, *β* = 0.59, *p*_FDR_ = 0.040), higher chitinase-3 like-1 (YKL-40, *β* = 0.73, *p*_FDR_ = 0.032), and lower interleukin-1beta (IL-1β, *β* = -0.67, *p*_FDR_ = 0.047) than those without HIV. There were no significant associations of any biomarkers with total PHQ-9 score. None of the indirect effects were significant, mediating <13.1% of the association. Findings remained consistent when accounting for age, gender, and time between neuroimaging and PHQ-9 administration. Using a robust analytical approach in a community-based sample, we have shown that participants living with HIV reported greater depressive symptoms than those without HIV, but we did not find that neuroimaging and blood biomarkers of inflammation significantly mediated this association. Further studies with participants experiencing severe depression may help to elucidate the links between HIV, inflammation, and depression.

## Introduction

People living with HIV are three times more likely to experience depressive symptoms compared to people without HIV [1]. In African countries, where the global majority of people living with HIV are located [2], 3.63 million people living with HIV were estimated to have major depressive disorder (MDD), resulting in loss of 1.57 million disability-adjusted life years [3]. The prevalence of suicide deaths amongst people living with HIV (10.2/1000 persons) is almost 100 times higher than the global incidence of suicide deaths in the general population (0.11/1000 persons) [4]. Young people (i.e. children and adolescents) living with HIV are similarly at elevated risk for depressive symptoms compared to young people without HIV [5, 6]. This risk is higher amongst women and girls, as well as those who experience stigma, food or housing insecurity, or other forms of social deprivation [7, 8]. Depression thus represents a substantial challenge for people living with HIV, especially for those who are socioeconomically marginalised.

Identifying mechanisms underlying this increased risk for depressive symptoms in people living with HIV may enable the development of effective interventions to treat depression in this community. In a subset of the general population (including young people), depression is associated with inflammation [9, 10]. People with depression exhibit increased concentrations of biomarkers of inflammation, including cytokines such as interleukin-6 (IL-6) and tumour necrosis factor-alpha (TNF-α) [11]. Early-life increases in the inflammatory biomarker IL-6 can predict the total number of depressive episodes later in life, even when accounting for gender, body mass index, and socioeconomic status [12]. People living with HIV exhibit chronic inflammation, evidenced by increased cytokine release and monocyte activation and alterations in neurometabolites such as choline (Cho) and myo-inositol (mI), which persist despite antiretroviral therapy (ART) [13–15]. Together, this evidence suggests that the increased risk for depression amongst people living with HIV may be driven by inflammation.

Inflammatory biomarkers which mediate a significant proportion of the association between HIV status and depressive symptoms may be useful targets for antidepressant interventions. The cytokines IL-6 and TNF-α have frequently been reported as being associated with depressive symptoms in people living with HIV [16]. In a recent study, we showed that controlling for the effects of certain cytokines and chemokines in blood and cerebrospinal fluid (CSF) attenuated the odds for depressive symptoms in people living with HIV by at least 10% [17]. These findings offered early evidence that these inflammatory biomarkers may at least partly mediate the association between HIV status and depressive symptoms. In the current study, we aimed to directly test whether inflammatory biomarkers mediate a significant proportion of this association in a sample of young people living with perinatally-acquired HIV and demographically comparable young people without HIV. We hypothesised that HIV status would be significantly associated with depressive symptoms, and that inflammatory biomarkers would significantly mediate this association.

## Materials and methods

### Participant recruitment

For this cross-sectional, observational study, participant recruitment was carried out between May and December 2022 at the Family Centre for Research with Ubuntu (FAMCRU) at Tygerberg Hospital in Cape Town, South Africa. We recruited a subset of participants from the Adolescent Cognitive Brain Imaging (GOLD) cohort. The GOLD cohort is an active longitudinal study investigating brain health and neurocognitive outcomes in adolescents living with perinatally-acquired HIV and demographically comparable adolescents without HIV, which followed on from the landmark Children with HIV Early antiretroviral (CHER) trial [18].

Participants were eligible for this study if they were younger than 18 years old and able to provide informed assent, with a parent or legal guardian able to provide informed consent. Participants living with HIV were eligible if they were currently receiving ART. To minimise selection bias, we did not target potential participants using previous history of depressive symptoms. Participants with a recent history of co-occurring infections (e.g. tuberculosis, HIV encephalopathy) were excluded from the study.

All participants completed a demographic questionnaire in their preferred language (English or isiXhosa). For participants living with HIV, we also received access to their clinical records, including the latest HIV viral load, CD4 cell counts (which are no longer routinely measured and thus not available for all participants), and ART. All participants were compensated for their travel expenses to attend the study visit.

This study was conducted in accordance with internationally recognised standards for ethical research and the International Conference on Harmonisation (ICH E6), and was approved by the Health Research Ethics Committee (HREC) of Stellenbosch University (N21/10/116_Sub Study N19/10/135) and the School of Psychology, Philosophy, and Language Sciences Research Ethics Committee (201-2122/3) of the University of Edinburgh. All participants provided written informed assent, and their parents or primary caregivers provided written informed consent, in the language of their choosing (English or isiXhosa).

### Patient Health Questionnaire (PHQ-9)

To quantify depressive symptoms, participants completed the nine-item Patient Health Questionnaire (PHQ-9) in their preferred language (English or isiXhosa). We developed the isiXhosa translation of the PHQ-9 using a transcultural translation framework for this study. This isiXhosa PHQ-9, the English-language version, and results from our validation study are available elsewhere [19]. Participants were provided a private space to complete the questionnaire, where they could complete the questionnaire themselves or ask for a trained member of the research staff to read out the questions and record their responses. Responses from the PHQ-9 were digitally captured following quality assurance checks. Any participants who scored >15 total or indicated suicide ideation on item 9 of the PHQ-9 were referred for further support to a qualified social worker or psychologist on staff.

### Neuroimaging biomarkers

To quantify neuroinflammation, participants underwent proton magnetic resonance spectroscopy (MRS). Our neuroimaging biomarkers of interest were choline-containing compounds (glycerophosphocholine + phosphocholine), which are associated with cell membrane turnover, and myo-inositol, which is associated with glial cell activation. Both choline and myo-inositol are used as biomarkers of neuroinflammation [20].

**Fig 1:**
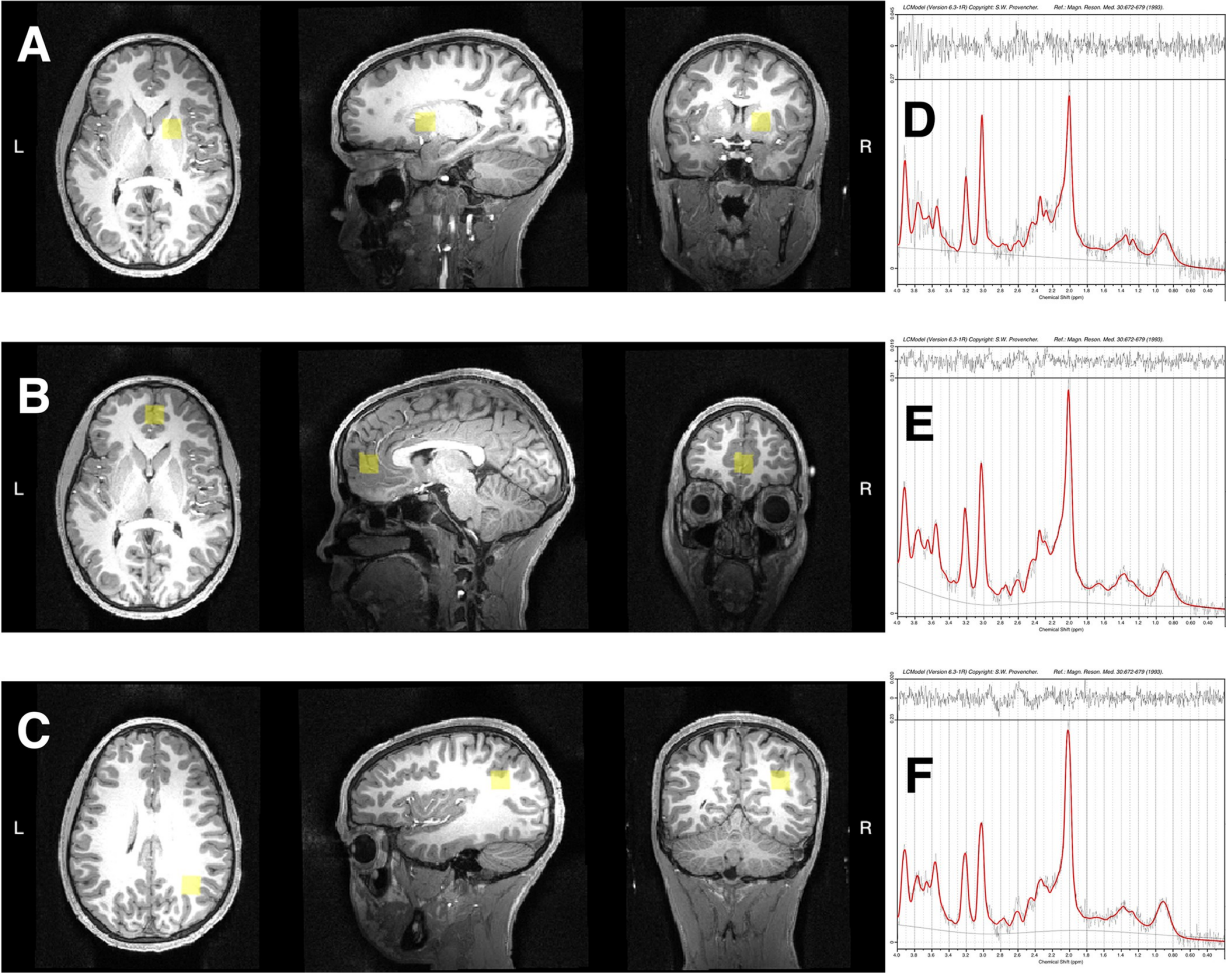
Voxels of interest and representative MRS spectra from one participant. **(A-C)** Positions of the voxels of interest (VOI, in yellow) in the (A) basal ganglia (BG), (B) midfrontal gray matter (MFGM), and (C) peritrigonal white matter (PWM), and **(D-F)** corresponding MRS spectra from the VOIs in (D) BG, (E) MFGM, and (F) PWM. Voxel placement images were produced using Gannet software: https://www.fil.ion.ucl.ac.uk/spm/software/spm12/.

Detailed methods for MRS image acquisition and processing for the GOLD cohort at age 11 have been reported elsewhere, and the same protocol was used in the current study [21]. Participants underwent scanning in a 3T Skyra scanner at the Cape Universities Body Imaging Centre (CUBIC) at the University of Cape Town. Single voxel MRS was acquired in three brain regions: basal ganglia (BG), midfrontal gray matter (MFGM), and peritrigonal white matter (PWM) (**Fig 1**). These brain regions were fixed by parent study design, and not necessarily selected for their association with depressive symptoms. Concentrations of choline and myo-inositol were determined in these regions as ratios to total water and to total creatine using LCModel version 6.3 via the method described previously [22]. Cases were excluded from analysis if any of the following quality control criteria were met: metabolite standard deviation (%SD) > 20%; full width at half maximum (FWHM) > 0.08; or signal-to-noise ratio (SNR) < 8. Apart from implementing these quality control criteria, we did not exclude any outliers from the MRS data.

### Blood biomarkers

To quantify peripheral inflammation, we collected blood samples from all participants at the same study visit during which the PHQ-9 was administered. Blood samples were allowed to clot, then centrifuged at 1500x g for 10 minutes to extract serum. Serum samples were frozen and stored at -80°C within 2 hours of collection.

Neurofilament light (NFL) concentrations in serum were determined using a human NFL enzyme-linked immunosorbent assay (ELISA) (BioTechne, Minneapolis, USA, catalogue number NDP2-81184). The assay was run according to manufacturer instructions in two replicates by two independent researchers with no sample dilution. One replicate failed to yield a satisfactory standard curve and was thus discarded. Absorbances from the NFL ELISA were converted to concentrations using a four parameter logistic (4-PL) curve.

Concentrations of remaining biomarkers of interest were determined using premixed human magnetic Luminex assays (BioTechne, Minneapolis, USA, catalogue number LXSAHM) analysed using a Luminex Bio-Plex 200 system (Bio-Rad, Hercules, USA). C-reactive protein (CRP) and soluble CD14 were detected using one 2-plex kit with a 1:200 sample dilution. Brain-derived neurotrophic factor (BDNF) and regulated upon activation normal T-cell expressed and secreted (RANTES/CCL5) were detected using one 2-plex kit with a 1:2 sample dilution. Soluble CD163, interleukin-1beta (IL-1β), IL-18, IL-6, interferon gamma-induced protein 10 (IP-10/CXCL10), monocyte chemoattractant protein 1 (MCP-1/CCL2), monokine induced by interferon gamma (MIG/CXCL9), macrophage inflammatory protein-1alpha (MIP-1α/CCL3), S100A8, tumour necrosis factor-alpha (TNF-α), and chitinase-3-like protein 1 (YKL-40/CHI3L1/HC gp-39) were detected using one 11-plex kit with a 1:2 sample dilution. All assays were run according to manufacturer instructions and in two replicates by two independent researchers. One replicate of the BDNF/RANTES assay and one replicate of the CRP/CD14 assay failed to yield a satisfactory standard curve and were thus discarded. Both replicates of the 11-plex assay yielded satisfactory standard curves.

Biomarker concentrations from Luminex assays were quantified by the instrument software (BioPlex Manager) using a five-parameter logistic (5-PL) curve. Where both replicates were quantified reliably, we averaged the two values. Where one replicate was quantified reliably and one was out-of-range (OOR) but a predicted value was computed, we retained only the reliably quantified replicate. Where both replicates were OOR but predicted values were available, we retained the replicate with the best-fitting standard curve, or if standard curves for both replicates were equally satisfactory, we averaged both predicted values. Where both replicates were below the detection range of the assay and no predicted values were computed, we assumed a value that was half the detection limit for that biomarker. Imputed values were assumed in this way for 36 cases for S100A8 and 5 cases for MIP-1α, but for no other biomarkers.

### Statistical approach

All analyses were conducted in R v.4.2.1. Demographic and HIV-related clinical characteristics were summarised using proportions (n and %) for categorical variables and medians and interquartile ranges (IQRs) for continuous variables. Normality of continuous variables was assessed using the Shapiro-Wilk test. Group differences between participants with and without HIV were assessed using Pearson’s chi-squared test or Fisher’s exact test (as appropriate) for categorical variables and Wilcoxon rank sum test for continuous variables.

There was no missingness in PHQ-9 data. After implementing exclusion criteria for the neuroimaging data, one case in the BG had a repeat measurement (carried out on the same day), from which the second measurement was retained and the first was excluded from analysis. In the PWM, seven cases had missing values, which were excluded from analysis.

In the Luminex immunoassays, data was missing for one participant for CRP and CD14, and one participant for BDNF and RANTES. These values were excluded from analysis. To detect outliers in the blood biomarker dataset, we identified values separately for each biomarker which were below the 1^st^ percentile or above the 99^th^ percentile for that biomarker. We then used the Rosner’s test for multiple outliers to test whether these values were true outliers; if so, these values were excluded. Outliers were excluded in this way for: BDNF (1 outlier), CD163 (1), CRP (1), IL-18 (2), IL-6 (1), IP-10 (2), MCP-1 (1), MIG (1), MIP-1α (1), and TNF-α (2).

Linear regression models were used to assess the main effect of HIV status on total PHQ-9 score for all participants, before and after adjusting for age (years, continuous) and self-identified gender (boy/girl).

**Fig 2:**
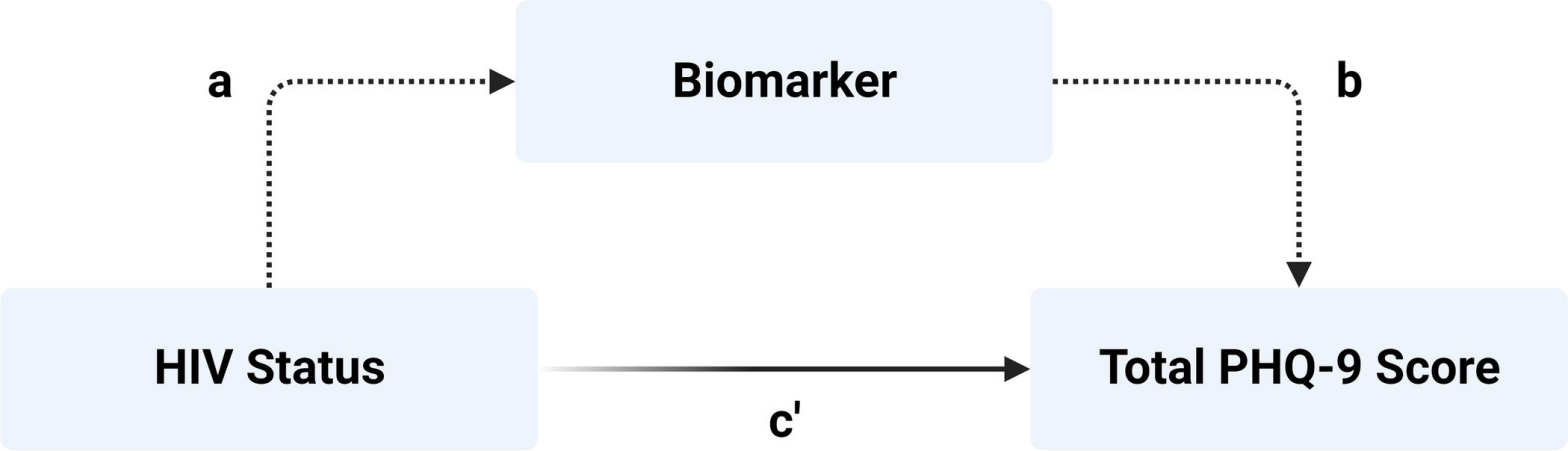
Path diagram for structural equation model (SEM). Our primary outcome of interest was the indirect effect (representing mediation–dotted lines) of each biomarker on the association between HIV status and total PHQ-9 score, indicated by path ab. The direct effect is represented by path c’.

Structural equation modelling (function sem in package lavaan v.0.6–16) was used to investigate the mediating effect of our biomarkers of interest on the main association between HIV status and total PHQ-9 score. The path diagram for the model is shown in **Fig 2**. Continuous variables (total PHQ-9 score, age, time difference between PHQ-9 and neuroimaging, and biomarker concentrations) were scaled before being input into models. For each biomarker separately, we determined standardised effect estimates with 95% confidence intervals (CIs) and *p* values using 1,000 bootstraps. Estimates were calculated for the direct effect of HIV status on total PHQ-9 score (c’ path), effect of HIV status on biomarker (a path), effect of biomarker on total PHQ-9 score (b path), total effect of HIV status on total PHQ-9 score, indirect effect of HIV status on total PHQ-9 score through the biomarker (ab path), and proportion of the association between HIV status and total PHQ-9 score mediated by the biomarker (expressed as % difference between the total effect and c’). Our primary outcome of interest was the indirect effect (ab path), which represents the mediation effect of the biomarker on the association between HIV status and depressive symptoms. Given that PHQ-9 scores were not normally distributed, we also ran SEM (adjusted for age and gender) with robust maximum likelihood estimation (“MLM”) for neuroimaging and blood biomarkers.

We also aimed to test the extent to which each biomarker might contribute unique, additive mediation effects beyond all other biomarkers, resulting in an aggregate increase in the proportion of the association between HIV status and depressive symptoms that could be explained by neuroimaging or blood biomarkers. We therefore ran multiple mediation SEM separately for neuroimaging and blood biomarkers, in which all biomarkers of the respective category were entered as simultaneous correlated mediators.

Statistical models were adjusted for age (years, continuous, scaled) and gender (boy/girl). Models involving neuroimaging biomarkers were also adjusted for the absolute time difference (days, continuous, scaled) between PHQ-9 administration and neuroimaging. All *p* values in SEM were corrected for multiple comparisons using the False Discovery Rate (FDR) method, and we report these FDR-corrected *q* values. We conducted a *post hoc* power analysis for our mediation effects using the Monte Carlo test in the pwrss shiny app v.0.3.1 (https://pwrss.shinyapps.io/index/) [23].

Finally, given the importance of assessing sex or gender differences to close the gender data gap in health research [24, 25], we also tested for differences in neuroimaging and blood biomarkers based on participants’ self-identified gender. Using linear regression models adjusted for age, we then explored whether there may be any gender differences in the mediating effects of inflammatory biomarkers on the association between HIV status and depressive symptoms.

## Results

### Participant characteristics

We recruited *N* = 60 participants, of whom *n* = 42 (70%) were participants living with HIV and *n* = 18 (30%) were participants without HIV. Summary demographic characteristics for participants are shown in **Table 1**. Participants were young people with a median (IQR) age of 15.5 (15.0, 16.0) years, relatively evenly split by gender (53% girls), and most participants self-identified as Black/African (92%) and heterosexual (92%). A modest proportion of participants reported alcohol use (25%) and a small proportion reported cigarette (6.7%) or recreational drug (12%) use in the preceding six months.

**Table 1:** Summary of demographic characteristics self-reported by participants, stratified by HIV status.

Characteristic	Overall *N* = 60	Participants	Participants	p-value
		with HIV	without HIV	
		*n* = 42	*n* = 18	
Age (years), Median (IQR) ^1^	15.5 (15.0, 16.0)	16.0 (15.0, 16.0)	15.0 (14.0, 15.0)	<0.001*
Gender, n (%) ^2^				0.4
Boy	28 (47%)	18 (43%)	10 (56%)	
Girl	32 (53%)	24 (57%)	8 (44%)	
Ethnicity, n (%) ^2^				0.3
Black/African	55 (92%)	37 (88%)	18 (100%)	
Coloured	5 (8.3%)	5 (12%)	0 (0%)	
Sexual orientation, n (%) ^2^				0.8
Bisexual/Pansexual	2 (3.3%)	1 (2.4%)	1 (5.6%)	
Gay/Lesbian/Homosexual	3 (5.0%)	2 (4.8%)	1 (5.6%)	
Straight/Heterosexual	55 (92%)	39 (93%)	16 (89%)	
Alcohol use, n (%) ^3^	15 (25%)	10 (24%)	5 (28%)	0.8
Cigarette smoking, n (%) ^3^	4 (6.7%)	4 (9.5%)	0 (0%)	0.3
Recreational drug use, n (%) ^3^	7 (12%)	6 (14%)	1 (5.6%)	0.7
Language of Administration, n (%) ^2^				0.2
English	13 (22%)	7 (17%)	6 (33%)	
isiXhosa	47 (78%)	35 (83%)	12 (67%)	

^1^ Wilcoxon rank sum test

^2^ Pearson’s Chi-squared test

^3^ Fisher’s exact test

* *p* < 0.05

Clinical characteristics of participants living with HIV are shown in **Table 2**. All participants living with HIV were on ART, with 90.5% being virally suppressed (i.e. with an HIV viral load <200 copies/mL at the most recent clinical evaluation). Participants with and without HIV were comparable on all demographic characteristics, except participants with HIV were older than those without HIV (*W* = 178, *p* < 0.001).

**Table 2:** Summary of clinical characteristics for participants living with HIV (*n* = 42).

Characteristic	Value
Viral Load < 200 copies/mL, n (%)	38 (90.5%)
CD4+ T-cell count (cells/μL), Median (IQR)	778 (625.75, 1011.75)
(Missing)	4
Antiretroviral Therapy Regimen, n (%)	
Tenofovir disoproxil fumarate + Lamivudine + Dolutegravir (TLD)	29 (69%)
Lopinavir + Ritonavir (LPV/r)	5 (12%)
Atazanavir	4 (9.5%)
Abacavir + Lamivudine (ABC/3TC)	1 (2.4%)
Dolutegravir	1 (2.4%)
Efavirenz (EFV)	1 (2.4%)
Emtricitabine + Tenofovir alafenamide (F/TAF)	1 (2.4%)

Median [IQR] total PHQ-9 score in the overall sample was 3 [0, 7]. Amongst participants living with HIV, median [IQR] total PHQ-9 score was 3.5 [0.25, 8], whereas amongst participants without HIV, median [IQR] total PHQ-9 score was 1.5 [0, 3]. Median [IQR] absolute time difference between PHQ-9 administration and neuroimaging was 92 [52, 130] days.

Number of participants for whom data was included for each biomarker is shown in **Table 3**.

**Table 3:** Biomarkers assessed in this study and number of participants for whom data was included for each biomarker.

Biomarker			Participants with HIV (*n*)	Participants without HIV (*n*)
*Neuroimaging biomarkers*	*Referencing*	*Region*		
Choline	Creatine Referenced	BG	34	17
		MFGM	37	18
		PWM	27	15
	Water Referenced	BG	34	17
		MFGM	37	18
		PWM	27	15
Myo-inositol	Creatine Referenced	BG	34	17
		MFGM	37	18
		PWM	27	15
	Water Referenced	BG	34	17
		MFGM	37	18
		PWM	27	15
*Blood biomarkers*				
BDNF			40	18
CD14			41	18
CD163			41	18
CRP			40	18
IL-1β			42	18
IL-18			40	18
IL-6			41	18
IP-10			40	18
MCP-1			41	18
MIG			40	18
MIP-1α			41	18
RANTES			41	18
S100A8			40	18
TNF-α			42	18
YKL-40			40	18

BDNF: brain-derived neurotrophic factor; BG: basal ganglia; CD: soluble cluster of differentiation; CRP: C-reactive protein; IL: interleukin; IP-10: interferon gamma-induced protein 10; MCP-1: monocyte chemoattractant protein 1; MFGM: midfrontal gray matter; MIG: monokine induced by interferon gamma; MIP-1α: macrophage inflammatory protein-1 alpha; PWM: peritrigonal white matter; RANTES: regulated upon activation normal T cell expressed and secreted; TNF-α: tumour necrosis factor alpha; YKL-40: chitinase-3 like-1 protein.

### Association between HIV status and depressive symptoms

**Fig 3:**
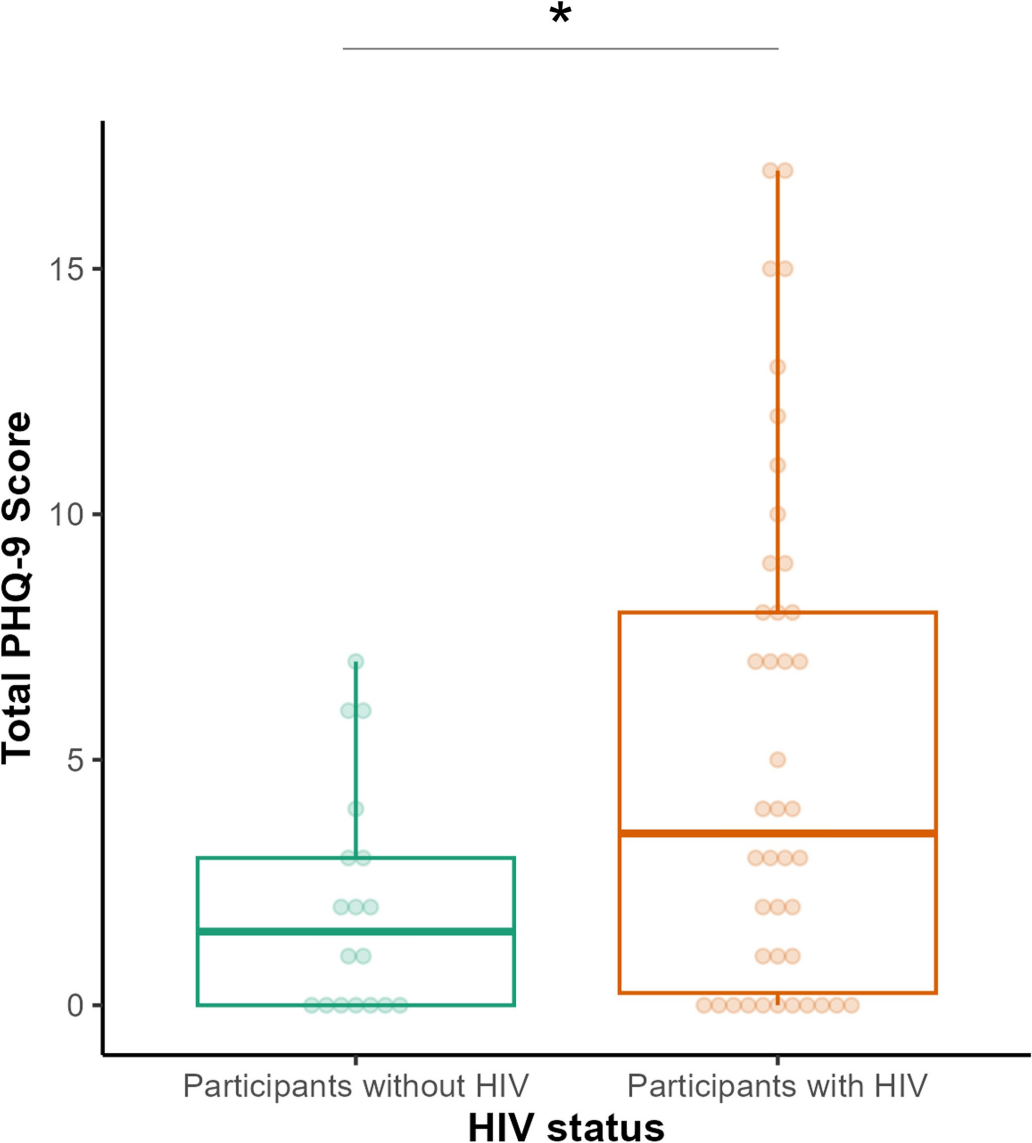
Self-reported depressive symptoms (quantified as total PHQ-9 score) for participants stratified by HIV status. The middle line in each box plot represents the median, with the upper and lower hinges representing the third and first quartile, respectively. * indicates p < 0.05.

We first assessed the direct effect of HIV status on depressive symptoms, quantified as total PHQ-9 score. There was a significant association between HIV status and depressive symptoms (*B* = 3.14, *β* = 0.66, *p* = 0.017), such that participants living with HIV reported higher severity of depressive symptoms than those without HIV (**Fig 3**). Findings remained consistent after adjusting for age and gender (*B* = 3.32, *β* = 0.70, *p* = 0.022).

### Correlations between inflammatory biomarkers

**Fig 4:**
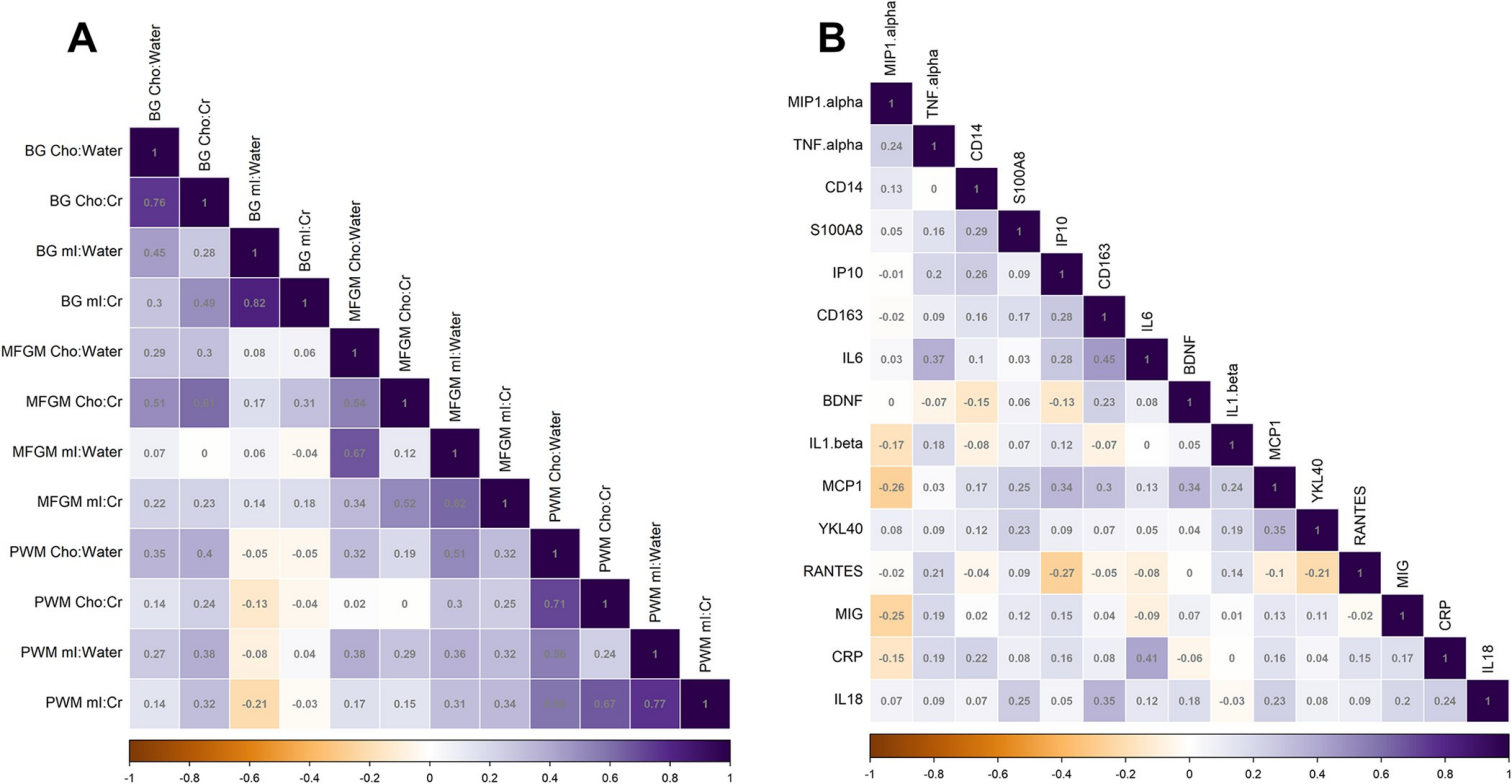
Spearman’s correlations (ρ) between **(A)** neuroimaging biomarkers and **(B)** blood biomarkers measured in this study. The strength of correlations is graded across a 3-point scale: −1.0 (in orange), 0.0 (in white), and +1.0 (in purple). For blood biomarkers, the correlation matrix is ordered by hierarchical clustering to optimise visual comparisons.

Concentrations of neuroimaging biomarkers were moderately correlated, with mean (standard deviation) absolute Spearman correlation of |*ρ*|  =  0.30 (0.21) and a range of −0.21 to 0.82 (**Fig 4A**). Modest correlations were observed for Cho:Cr between the BG and MFGM (*ρ* = 0.61), Cho and mI referenced to water in the MFGM (*ρ* = 0.67), and Cho and mI referenced to creatine in the PWM (*ρ* = 0.67).

Concentrations of blood biomarkers were only weakly or moderately correlated, if at all, with mean (standard deviation) absolute Spearman correlation of |*ρ*|  =  0.14 (0.10) and a range of −0.27 to 0.45 (**Fig 4B**). The strongest observed correlations, which were still only moderate, were between IL-6 and soluble CD163 (*ρ* = 0.45) and IL-6 and CRP (*ρ* = 0.41).

Biomarker correlations separately for participants with and without HIV, as well as correlations between all neuroimaging and blood biomarkers, are available in **S1 Fig**.

### Neuroimaging biomarkers as mediators of the HIV-depression association

**Fig 5:**
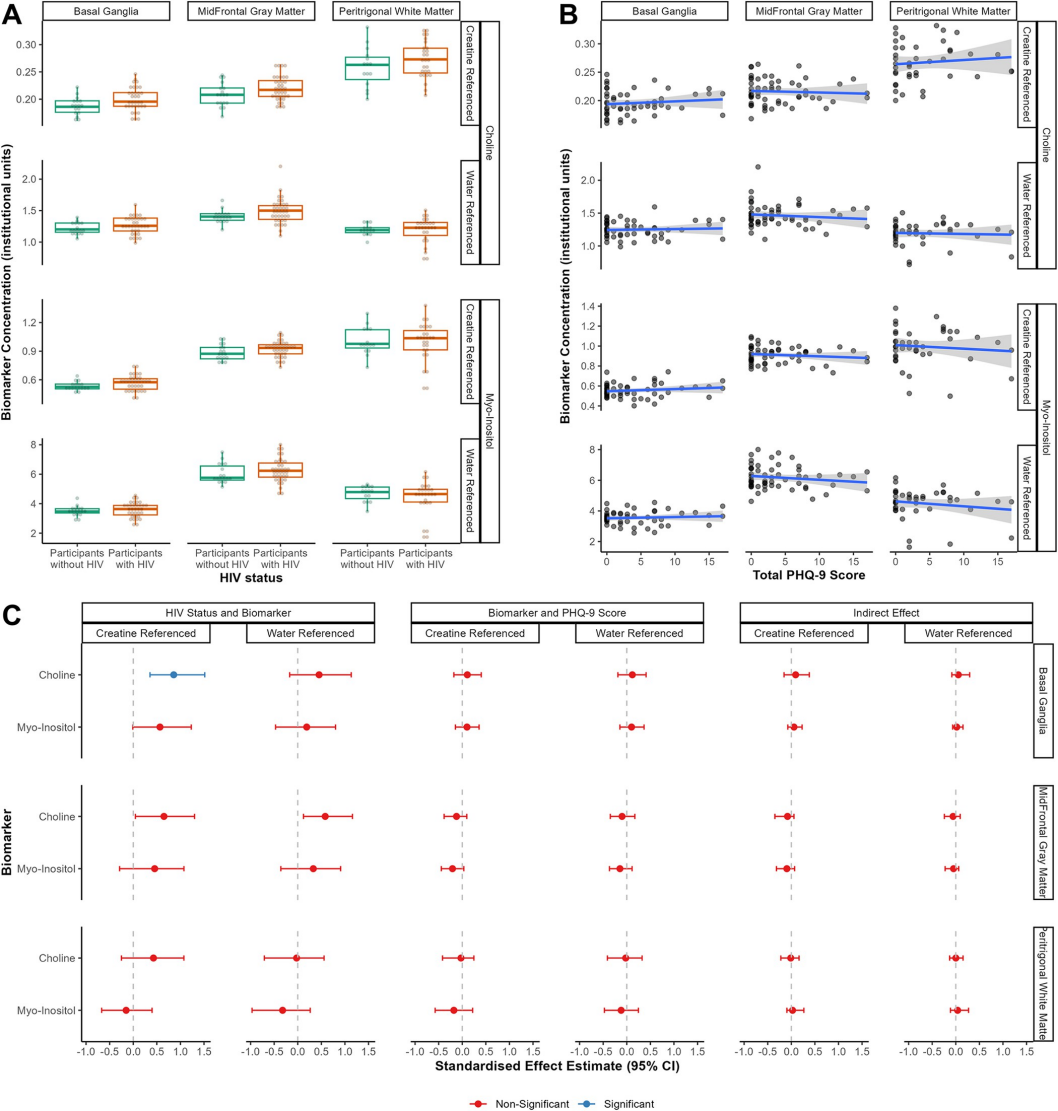
Concentration (institutional units) of choline and myo-inositol, across two referencing protocols and three brain regions, by **(A)** HIV status, and **(B)** total PHQ-9 score. Values shown in (A) and (B) are unadjusted for covariates. **(C)** Standardised effect estimates with 95% confidence intervals (CI) for structural equation modelling (SEM) involving the neurometabolites choline and myo-inositol across two referencing protocols and three brain regions, adjusted for age, gender, and absolute time difference (in days) between PHQ-9 administration and neuroimaging. Path estimates which are significantly different from zero (after correcting p values for multiple comparisons) are shown in blue.

Concentrations of choline and myo-inositol (referenced to creatine and water) in each brain region stratified by HIV status and as a function of total PHQ-9 score are shown in **Fig 5A** and **5B**. Mean concentrations (ratio) and standard deviation (SD) for each biomarker, referencing protocol, and brain region is available in **S1 Table**.

In SEM adjusted for age, gender, and absolute time difference in days between PHQ-9 administration and neuroimaging (**Fig 5C**), there was a significant association between HIV status and choline referenced to creatine in the basal ganglia, *β* (95% CI) = 0.86 (0.36, 1.52), *q* = 0.035. Participants with HIV thus showed a higher choline-to-creatine ratio in the BG than those without HIV. There were no other significant associations between HIV status and the neuroimaging biomarkers, and no significant associations between these biomarkers and total PHQ-9 score (all other *β* < |0.65|, *q* > 0.05). Indirect effect estimates for these biomarkers were not significant, mediating <13.1% of the association between HIV status and total PHQ-9 score (all *β* < |0.09|, *q* > 0.05).

We ran multiple mediation SEM in which all six MRS biomarkers (choline and myo-inositol in the BG, MFGM, and PWM each) were entered as simultaneous correlated mediators, separately for biomarkers referenced to creatine and to water. Compared to the largest mediation signals of individual neuroimaging biomarkers (13.1% for creatine-referenced choline in the BG and 12.7% for creatine-referenced myo-inositol in the MFGM), all creatine-referenced neuroimaging biomarkers together mediated 46.32% of the association between HIV status and depressive symptoms, though this mediation was also non-significant (indirect effect *β* (95% CI) = 0.31 (-0.29, 1.28), *q* = 0.77). Similarly, all water-referenced neuroimaging biomarkers together mediated 9.22% of this association, and this mediation was also non-significant (indirect effect *β* (95% CI) = 0.06 (-0.38, 0.90), *q* = 0.99).

### Blood biomarkers as mediators of the HIV-depression association

NFL was undetectable in our human NFL ELISA for all except two participants. We therefore did not carry out any further statistical analyses with NFL data.

**Fig 6:**
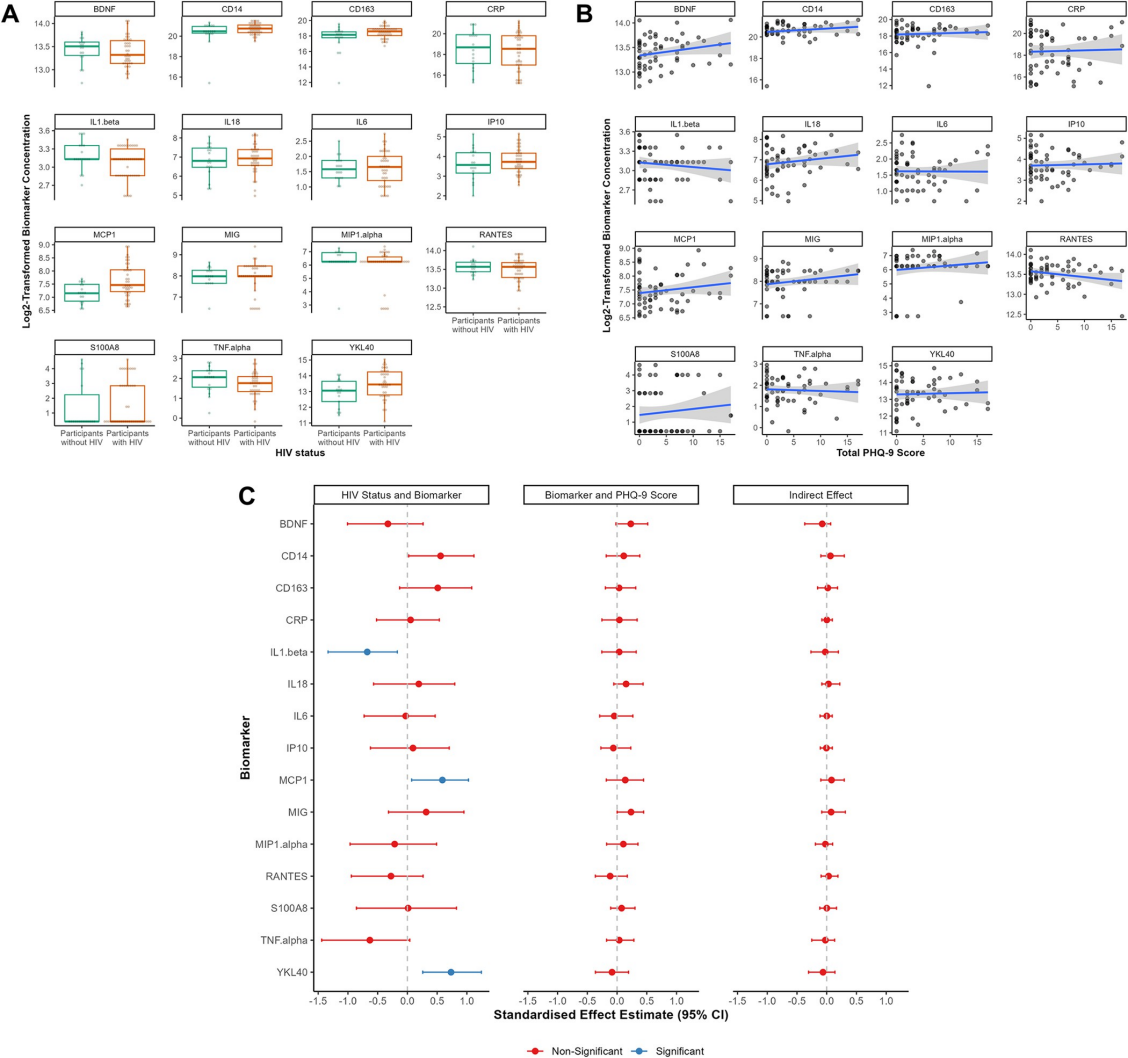
Concentration (log2-transformed) of blood biomarkers by **(A)** HIV status, and **(B)** total PHQ-9 score. Values shown in (A) and (B) are unadjusted for covariates. **(C)** Standardised effect estimates with 95% confidence intervals (CI) for structural equation modelling (SEM) involving biomarkers quantified in blood serum, adjusted for age and gender. Path estimates which are significantly different from zero (after correcting p values for multiple comparisons) are shown in blue.

Concentrations of blood biomarkers by HIV status and total PHQ-9 score are shown in **Fig 6A** and **6B**. Mean (SD) absolute concentrations (pg/mL) for each biomarker are available in **S2 Table**.

In SEM adjusted for age and gender (**Fig 6C**), there was a significant association between HIV status and IL-1β, *β* (95% CI) = -0.67 (-1.33, -0.17), *q* = 0.047, such that participants with HIV showed lower concentrations of IL-1β than those without HIV. There were also significant associations between HIV status and MCP-1, *β* (95% CI) = 0.59 (0.07, 1.03), *q* = 0.040 and YKL-40, *β* (95% CI) = 0.73 (0.26, 1.24), *q* = 0.003, such that participants with HIV showed higher concentrations of these biomarkers than those without HIV. There were no other significant associations between HIV status and blood biomarkers measured in this study, and no significant associations of these biomarkers with total PHQ-9 score (all other *β* < |0.63|, *q* > 0.05). Indirect effect estimates for these biomarkers were not significant, mediating <11.0% of the association between HIV status and total PHQ-9 score (all *β* < |0.08|, *q* > 0.05).

Given the modest correlations across blood biomarkers, we ran a multiple mediation SEM in which all 15 blood biomarkers were entered as simultaneous correlated mediators. Compared to the largest mediation signal of individual blood biomarkers (11% for MCP-1 and 10.5% for BDNF), all blood biomarkers together mediated 16.25% of the association between HIV status and depressive symptoms, though this mediation was also non-significant (indirect effect *β* (95% CI) = -0.11 (-0.98, 0.66), *q* = 0.99).

Unadjusted SEM analyses for neuroimaging and blood biomarkers are available in **S2–S3 Figs**. Findings were consistent across unadjusted and adjusted analyses for both neuroimaging and blood biomarkers, except that the association between IL-1β and HIV status was not significant before adjusting for age and gender, *β* (95% CI) = -0.54 (-1.12, -0.03), *q* = 0.130. All path estimates, 95% CIs, and *p* values for our SEM analyses (unadjusted, adjusted, and only including participants with HIV who were virally suppressed) are available in **S3 Table**. Findings remained consistent when excluding the *n* = 4 participants with HIV who had an HIV viral load >200 copies/mL. We also ran SEM adjusted for age and gender with the MLM estimator to account for non-normality in the outcome (PHQ-9 scores). Effect sizes and confidence intervals for all path estimates were largely consistent between the two sets of analyses, although path estimates for blood biomarkers did not survive FDR correction in models with the MLM estimator (**S4 Table**).

A *post hoc* power analysis for the largest absolute mediation effect in our findings (*β* = -0.09 for creatine-referenced myo-inositol in the MFGM) revealed that this effect was detected with 31% power.

There were several nominally significant differences in neuroimaging and blood biomarkers differed based on participants’ gender, including water-referenced choline in the basal ganglia and IL-1β and YKL-40 in serum (though these associations did not survive FDR correction; **S4 Fig**). We then tested whether there may be any gender differences in the mediating effects of inflammatory biomarkers on the association between HIV status and depressive symptoms. We found no significant interactions between gender and HIV status on biomarker concentrations or between gender and biomarker concentrations on total PHQ-9 score (**S5 Table**). Given this lack of significant gender interactions in path a and path b of our mediation model, we did not further assess the interaction of gender with our indirect effect of interest.

## Discussion

In a sample of young people living with and without HIV, we found that HIV status is associated with depressive symptoms. When accounting for age and gender, participants with HIV on average scored 3.32 points higher on the PHQ-9 compared to participants without HIV. Participants with HIV also exhibited significantly lower IL-1β but higher MCP-1 and YKL-40 concentrations in blood serum. However, associations between biomarkers (in blood or brain) and PHQ-9 were entirely null. Thus, we did not find support for our hypothesis that inflammatory biomarkers mediate the association between HIV status and depressive symptoms: none of the neuroimaging or blood serum biomarkers measured in our study mediated a significant proportion of this association.

After adjusting for age and gender, one neuroimaging biomarker (choline referenced to creatine in the basal ganglia) and three inflammatory biomarkers in blood serum (IL-1β, MCP-1, and YKL-40) were significantly associated with HIV status. We have previously observed higher choline levels in basal ganglia of children with HIV compared to those without HIV in the CHER cohort, and a similar association was observed in the current study [26]. However, as this effect was not observed for choline referenced to water, it is possible that this association was confounded by alterations in creatine concentrations. Moreover, alterations in choline and myo-inositol concentrations may be driven by mechanisms other than neuroinflammation, such as alcohol use [27]. Concentrations of choline may also be influenced by other physiological processes which impact cell membrane turnover, including myelination, which occurs rapidly during adolescence [28, 29]. Therefore, the lack of associations between HIV status and neurometabolites measured in this study may be partly attributed to possible confounding mechanisms and the non-specific nature of these biomarkers.

In alignment with existing evidence, we saw that participants with HIV exhibited higher MCP-1 than those without HIV [30]. We also found that participants with HIV showed higher YKL-40 compared to those without HIV. YKL-40 is a relatively novel marker associated with astrocytic activation and neurodegeneration [31]. Few studies to date have compared YKL-40 concentrations between people with and without HIV. Hermansson et al. [32] found that YKL-40 concentrations in CSF did not differ between participants with and without HIV. Guha and colleagues [33] recently also observed no significant difference in plasma or CSF concentrations of YKL-40 by HIV status, but significantly higher YKL-40 in CSF (compared to people without HIV) specifically in people with HIV with a CSF HIV viral load higher than 50 copies/mL [34]. Notably, these studies were carried out in older adults. We report the first evidence that YKL-40 in blood serum is significantly higher (*β* = 0.73) in young people living with perinatally-acquired HIV compared to demographically comparable participants without HIV. This difference in serum YKL-40 may be indicative of ongoing astrocytic activation and neuronal injury in participants with HIV despite suppressive ART.

We observed that IL-1β was significantly lower in participants with HIV compared to participants without HIV. Absolute differences in median IL-1β concentration between participants with and without HIV were small (8.43 pg/mL vs 9.25 pg/mL respectively). However, the strength of association between HIV status and IL-1β was fairly large, both before (β = -0.54) and after (β = -0.67) adjusting for age and gender. This finding is unexpected, as previous studies have generally observed higher IL-1β in serum of participants with HIV, including those who are virally suppressed [35, 36]. Notably, at least one study has reported lower IL-1β in participants with HIV compared to those without HIV, but this measurement was in cervicovaginal lavage and not in blood [37]. IL-1β and IL-18 are both secreted as a result of inflammasome activation, and thus are expected to be correlated [38]. However, in our sample, there was almost no correlation between IL-1β and IL-18 (Spearman’s *ρ* = -0.03), which suggests possible ‘masking’ of IL-1β concentrations. One potential explanation for lower IL-1β observed in participants with HIV may be higher levels of receptor expression on certain cells in these participants, leading to receptor binding of the cytokine and thus removal of IL-1β from the serum. However, this is speculative given that many previous studies have observed higher IL-1β in participants with HIV, and future replication of these findings is necessary to clarify the direction of the association between HIV status and IL-1β.

Although we observed HIV-related differences in concentrations of certain neuroimaging and blood biomarkers, none of the biomarkers measured in this study were significantly associated with total PHQ-9 score. For neuroimaging biomarkers, this may be in part because the brain regions in which these metabolites were measured in this study were not necessarily selected for their importance in the pathogenesis of depression. Previous findings have suggested that depressive symptoms may be associated with alterations in choline concentrations in the prefrontal cortex and putamen, or myo-inositol concentrations in the anterior cingulate cortex and prefrontal cortex [39]. It will therefore be important to examine these brain regions in future studies of depressive symptoms in young people with HIV. Furthermore, although several previous studies have observed significant associations between inflammatory biomarkers and depressive symptoms in people with HIV, this research has largely been carried out in adults with HIV acquired later in life [16], whereas our study focused on young people living with perinatally-acquired HIV. Since perinatal HIV infection in the brain results in distinct and persistent alterations in white matter microstructure [40–42], it is possible that depressive symptoms in our participant sample were driven by these neuroanatomical changes rather than neurometabolite concentrations.

Soluble inflammatory biomarkers such as IL-6 and TNF-α have been frequently observed to be associated with depressive symptoms in other studies, so the lack of statistically significant associations in our sample was surprising [16]. We recruited a community-based sample and thus observed a ‘natural’ spread of depressive symptoms in our sample, with a few participants reporting severe depressive symptoms, while the majority reported no or low depressive symptom severity (median PHQ-9 score of 3). This prevalence of depressive symptoms is comparable with previous studies; for instance, in a larger sample of adults with HIV in which we recently showed a potential mediating role for inflammatory biomarkers, we observed a median [IQR] PHQ-9 score of 2 [0, 4] [17]. Nevertheless, the relatively low prevalence of depressive symptoms in our sample may explain why we did not detect any significant associations with inflammatory biomarker concentrations, and, by extension, why we did not find any significant mediation by inflammatory biomarkers of the association between HIV status and depressive symptoms.

Certain limitations of the current study are noted. We had a small sample size (*N* = 60), which may have limited our ability to reliably detect true underlying associations, as demonstrated by the post hoc power analysis which indicated that we could detect the largest mediation effect in our study with only 31% power [43]. We only measured inflammation and depression at a single time-point, thus no inferences can be made about the temporality of the associations between these variables. Additionally, as the PHQ-9 is a screening (not diagnostic) tool, measurement of depressive symptoms using this tool instead of a more comprehensive interview may also have influenced our findings. Although we endeavoured to schedule participant study visits close to the neuroimaging visits, in practice, MRS acquisition and PHQ-9 administration were separated by about 3 months for most participants. This time difference limits our interpretation of the association between PHQ-9 score and neuroimaging biomarkers of inflammation, since depressive symptoms can change transiently even over the course of a few weeks [44]. However, we aimed to partly mitigate this limitation by including this time difference as a statistical covariate in our analysis. Concentrations of inflammatory biomarkers in blood, and NFL in particular, may be measured by more sensitive immunoassays such as single molecule array (SIMOA). Finally, while we controlled for the effects of age and gender in our analyses, our findings may have been influenced by other potential confounders such as socioeconomic status or antiretroviral regimens, which can also impact inflammation and risk for depression [45, 46].

Despite these limitations, our study offers some important advantages. Research on the mental health of people living with HIV has relied on primarily White, primarily male samples, with many studies in the field involving up to 90% White and male participants [47]. Our study adds much-needed diversity to this field, with our participant sample being primarily Black/African and gender-diverse (53% girls). Our participant sample is comparable to the population of young people living with HIV in South Africa and globally, as the majority of these young people are Black/African and girls [48, 49]. Previous research has largely focused on describing associations between inflammatory biomarkers and depressive symptoms. We take this work one crucial step further by explicitly assessing whether inflammatory biomarkers mediate depressive symptoms in people with HIV. Identifying biomarkers which mediate this association will support the development of diagnostic or therapeutic targets for depression in this community. We used structural equation modelling (a robust approach to mediation analysis) to investigate this question, with a rigorous analytical pipeline which accounted for multiple comparisons and key sociodemographic factors. Finally, we assessed the mediating role of inflammation using multiple modalities: neurometabolites (with two separate referencing protocols) and blood serum proteins (measured using latest-generation high-sensitivity immunoassays), lending further robustness to our null findings.

We found no evidence that inflammatory biomarkers significantly mediated the association between HIV status and depressive symptoms in a sample of young people. Future studies may seek to explore the associations we observed between inflammatory biomarkers and HIV status, particularly those for IL-1β and YKL-40, and investigate these mediation effects in larger samples. Studies focusing on participants with severe depressive symptoms may help to clarify whether inflammatory biomarkers mediate a significant proportion of the association between HIV and clinical depression. Longitudinal studies tracking the trajectories of depressive symptoms and inflammatory biomarkers in young people with HIV may enable us to determine whether inflammation predicts depression or vice versa in this population. Our sample comprised young people living with perinatally-acquired HIV who were initiated on ART early. Future work may thus seek to determine whether early (versus delayed) ART initiation plays a role in dampening any associations between inflammation and depressive symptoms in young people with HIV. Identifying significant neurobiological mediators of the association between HIV status and depression may reveal useful therapeutic targets. Nevertheless, the contributions of antiretroviral medication and psychosocial factors such as stigma, discrimination, or socioeconomic adversity must be given equal consideration when exploring possible mechanisms driving the risk for depression in people with HIV.

## Supporting information

S1 Fig Spearman’s correlations between neuroimaging biomarkers, blood biomarkers, and all biomarkers separately for participants with and without HIV. (TIF)

S2 Fig Unadjusted standardised effect estimates for SEM involving neuroimaging biomarkers. (TIF)

S3 Fig Unadjusted standardised effect estimates for SEM involving blood biomarkers. (TIF)

S4 Fig Unadjusted standardised effect estimates for associations of all biomarkers with participant age, gender, and HIV status. (TIF)

S1 Table Summary statistics for neuroimaging biomarkers. (XLSX)

S2 Table Summary statistics for blood biomarkers. (XLSX)

S3 Table All path estimates for SEM (adjusted and unadjusted) for neuroimaging and blood biomarkers. (XLSX)

S4 Table All path estimates for SEM (adjusted) for neuroimaging and blood biomarkers using robust maximum likelihood estimation (MLM). (XLSX)

S5 Table Standardised effect estimates for interactions between gender and HIV status on biomarker concentrations or between gender and biomarker concentrations on total PHQ-9 score. (XLSX)
