# Supplementary figures and images for: Multi-modal analysis of inflammation as a potential mediator of depressive symptoms in young people with HIV: The GOLD depression study
Source: PLoS One. 2024 Feb 22;19(2):e0298787. doi: 10.1371/journal.pone.0298787 (PMC10883559; doi:10.1371/journal.pone.0298787)

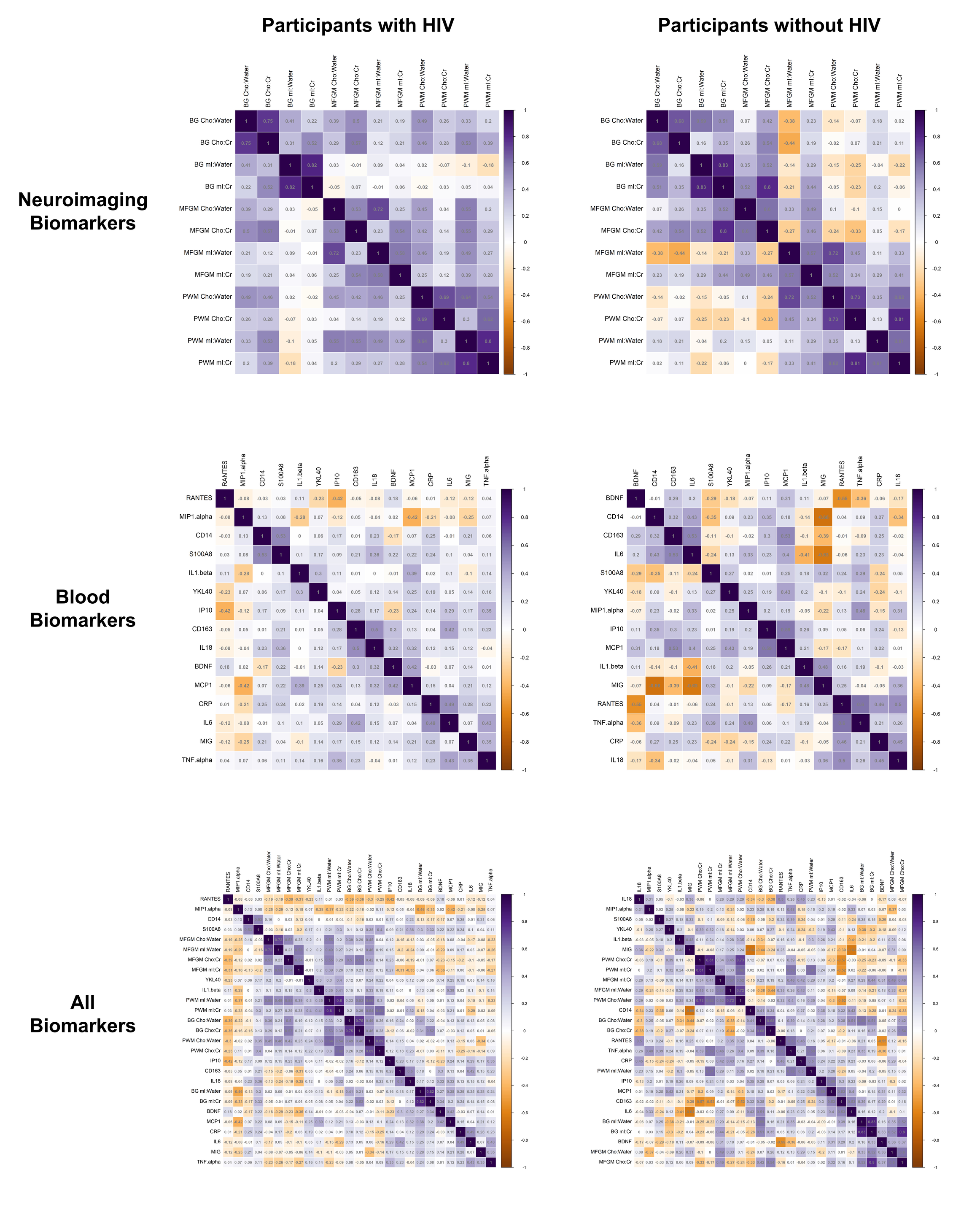

Supplement: S1 Fig — (TIF) [file pone.0298787.s001.tif]

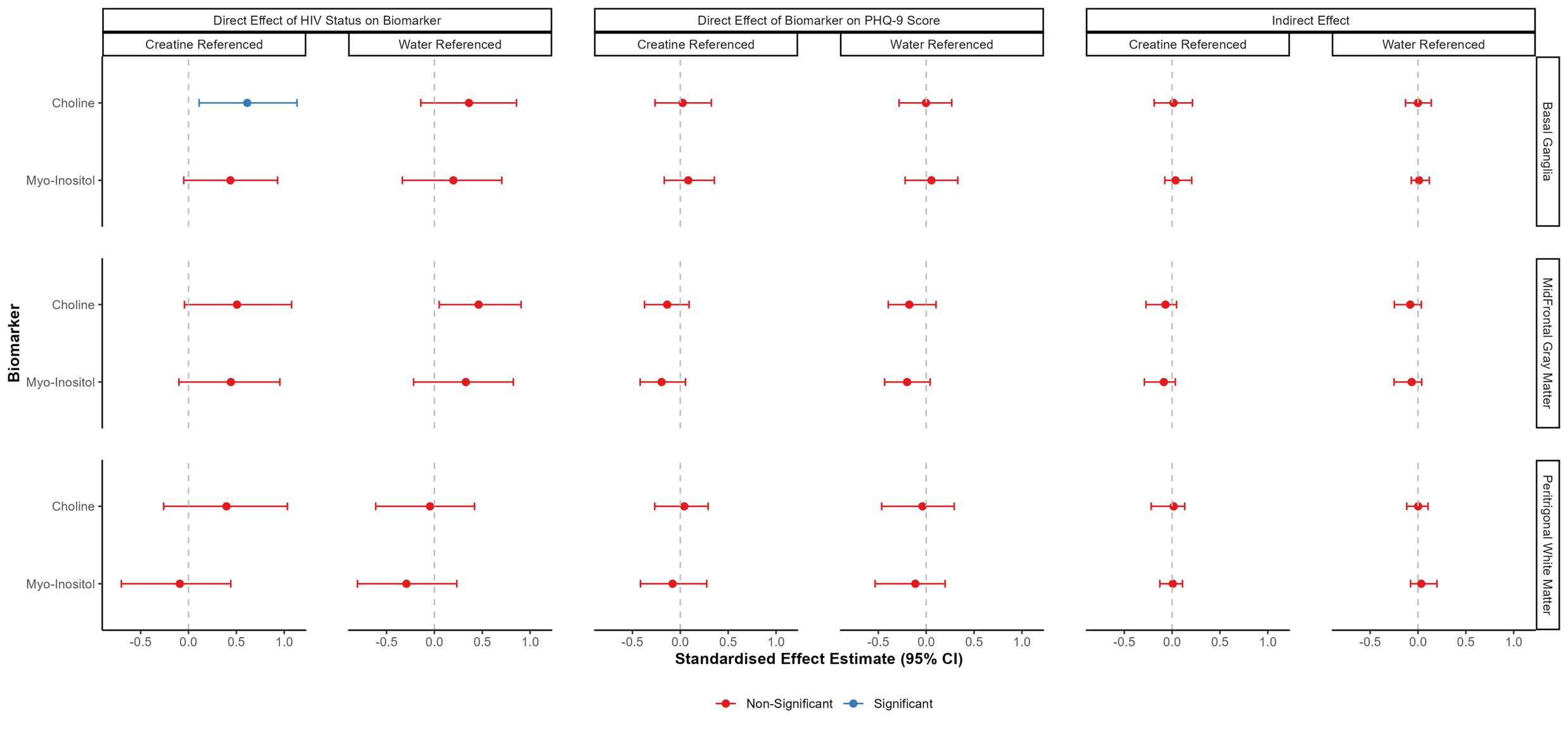

Supplement: S2 Fig — (TIF) [file pone.0298787.s002.tif]

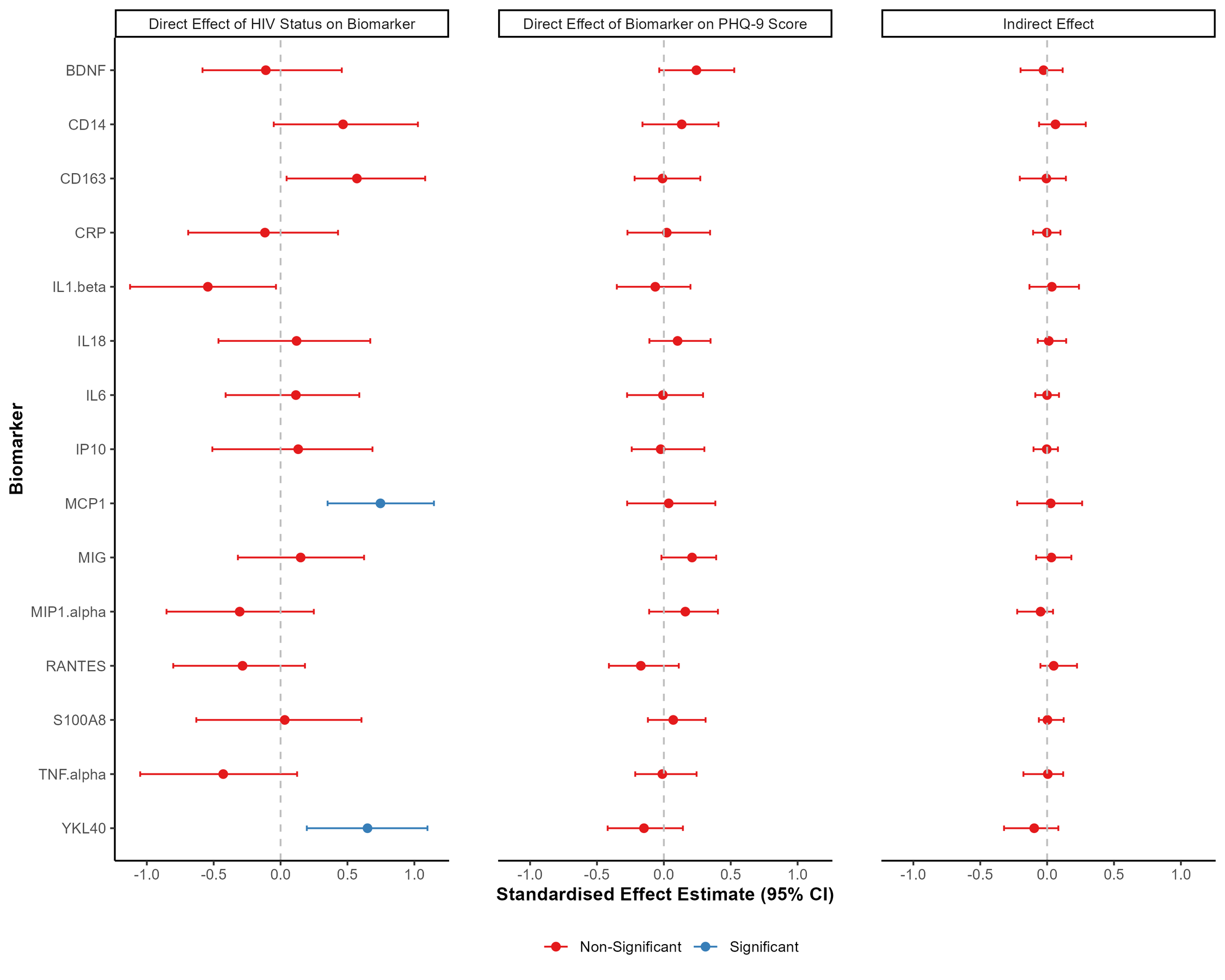

Supplement: S3 Fig — (TIF) [file pone.0298787.s003.tif]

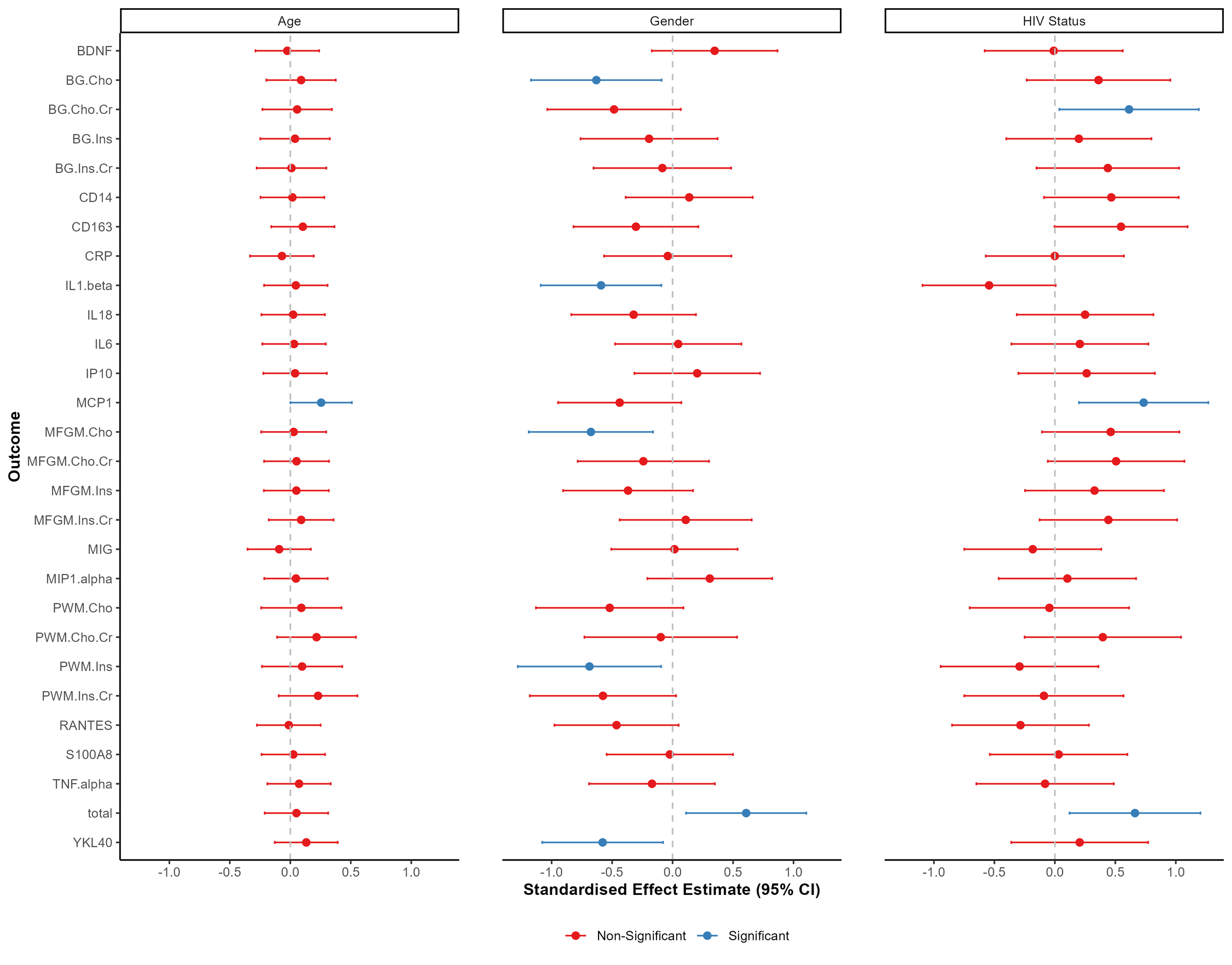

Supplement: S4 Fig — (TIF) [file pone.0298787.s004.tif]
